# Nomograms of Iranian fetal middle cerebral artery Doppler waveforms and uniformity of their pattern with other populations' nomograms

**DOI:** 10.1186/1471-2393-8-50

**Published:** 2008-11-12

**Authors:** Mohammad Kazem Tarzamni, Nariman Nezami, Narges Sobhani, Nazanin Eshraghi, Maryam Tarzamni, Yashar Talebi

**Affiliations:** 1 Department of Radiology, Tabriz University of Medical Sciences, Tabriz, Iran; 2 Young Researchers Club, Tabriz Islamic Azad University, Tabriz, Iran; 3 Drug Applied Research Center, Tabriz University of Medical Sciences, Tabriz, Iran; 4 Obstetrics and Gynecology ward, 29 Bahman Hospital, Tabriz, Iran

## Abstract

**Background:**

Doppler flow velocity waveform analysis of fetal vessels is one of the main methods for evaluating fetus health before labor. Doppler waves of middle cerebral artery (MCA) can predict most of the at risk fetuses in high risk pregnancies. In this study, we tried to obtain normal values and their nomograms during pregnancy for Doppler flow velocity indices of MCA in 20 – 40 weeks of normal pregnancies in Iranian population and compare their pattern with other countries' nomograms.

**Methods:**

During present descriptive cross-sectional study, 1037 normal pregnant women with 20^th^–40^th ^week gestational age were underwent MCA Doppler study. All cases were studied by gray scale ultrasonography initially and Doppler of MCA afterward. Resistive Index (RI), Pulsative Index (PI), Systolic/Diastolic ratio (S/D ratio), and Peak Systolic Velocity (PSV) values of MCA were determined for all of the subjects.

**Results:**

Results of present study showed that RI, PI, S/D ratio values of MCA decreased with parabolic pattern and PSV value increased with simple pattern, as gestational age progressed. These changes were statistically significant (P = 0.000 for all of indices) and more characteristic during late weeks of pregnancy.

**Conclusion:**

Values of RI, PI and S/D ratio indices reduced toward the end of pregnancy, but PSV increased. Despite the trivial difference, nomograms of various Doppler indices in present study have similar pattern with other studies.

## Background

Although for the first time, Satomura demonstrated that the Doppler ultrasonography (DU) technique could evaluate blood flow, but more than two decades passed until this technique was utilized in the assessment of fetal haemodynamics [1]. Currently, DU velocimetry of uteroplacental, umbilical and fetal vessels has become established method of antenatal monitoring, allowing the non-invasive assessment of fetal circulation [2] and its indices provide important information on the haemodynamics of the vascular area under study [3] that were not readily obtained from more conventional tests of fetal well-being [4,5]. Circulatory changes, reflected in certain fetal Doppler waveforms, predict adverse perinatal outcome [6,7]. In experienced hands, waveforms from a number of fetal vessels predict the occurrence and timing of adverse events.

Although umbilical arteries are the common vessels assessed by DU, recent studies confirm the efficacy of middle cerebral artery (MCA) Doppler assessment and advocate it [8,9]. Moreover, it has been showed that evaluation of MCA flow velocitometry could provides information about other fetal organs perfusion [10]. So, The MCA has emerged as the vessel of choice in Doppler assessment of fetal intracranial and other organs perfusion, because of its highest resistance indices and earlier presentation of diastolic blood flow, improved ultrasound resolution, advanced pulsed and color coded DU [11,12].

MCA Doppler measurement is a well-known modality for detecting fetal compromise [13]. Some studies showed that MCA blood flow abnormalities were associated with hypoxia [1,9,14], adverse perinatal outcome [15] and suboptimal neurodevelopment [16]. Thus, its evaluation by pulsed Doppler has become standard for antenatal care of high-risk pregnancies such as those suspected to affect by growth restriction [17], multiple pregnancies [18], pregnancy-induced hypertension [19], fetal parvovirus B19 infection [20], fetal anemia [17], Rh immunization and hydrops fetalis [8], fetal malformations [21] and large chorioangioma [22].

Because of the potential role of reference range for diagnosis of abnormal condition, reference values for indices derived from the flow velocity waveforms of several vessels of fetus in uncomplicated pregnancies have previously been recorded [23-25]. Therefore, we aimed to determine and establish Doppler indices nomograms of the fetal MCA for our own antenatal population and compare these findings with other authors reports.

## Methods

This study was performed as a prospective cross-sectional (February 2004 to May 2007) analysis of Doppler measurements performed between 20 and 40 weeks of gestation in 1037 low risk pregnancies. A power of 90% (β = .10) and significancy of 5% were applied to calculate sample size of 20 cases per each week of gestational age [n = (z*sd/d)^2^], but we enrolled more than 40 cases for each gestational week. All of these women were from a low-risk population and referred to receive routine prenatal care at our department. The research protocol was approved by the local ethics committee and written informed consent was obtained from every patient involved in the study prior to the examination.

Prior to Doppler assessment, initially all pregnant women underwent gray scale ultrasonography to evaluate multiple pregnancies, fetal anatomy and biophysical profile (the latter according to Manning's criteria [26]) including abdominal circumference (AC), head circumference (HC), femur length (FL), and biparietal diameter (BPD). Estimated fetal weight was calculated according to the Shepard and Hadlock formulas. Furthermore, amniotic fluid volume and placental grade were evaluated.

Doppler examinations were performed by a single investigator in Alzahra Obstetrics & Gynecology Hospital using Hitachi model EUB 525 (Hitachi Medical Corp, Tokyo, Japan) by 3.5 MHz convex transducer. Before beginning MCA assessment, a uterine artery Doppler waveform was obtained and then Doppler examinations were performed on the woman placed in a recumbent or semirecumbent position. The high-pass filter was set at 50–100 Hz in both imaging and Doppler modes to eliminate signals from slowly moving organs. The power setting was <50 mW/cm2 spatial temporary average velocity in both imaging and Doppler modes and the sample volume was 2–3 mm for the MCA. The scanning plane was adjusted to obtain an insonation angle as close as possible to 0°, and always<20°. During periods of stopped fetal breathing and movements, the image was frozen and the waveforms were quantified. Attention was taken to avoid any unnecessary pressure on the fetal head and the mechanical and thermal indices were always kept below 1. Every patient underwent only one examination. To measure the MCA, an ultrasound scan of the fetal head was performed to obtain a transverse view at the level used usually to measure BPD. The transducer was then moved parallel to this plane towards the base of the skull at the level of the lesser wing of the sphenoid bone to identify the circle of Willis. At the level of the lesser wing of the sphenoid bone, the MCA is easily demonstrated as a major branch of the circle of Willis. After localization of the MCA by color Doppler flow, velocity was measured from proximal portion of MCA. When the best quality was obtained for flow velocity waveforms, at least three waveforms were measured by sonologist and averaged. Multiple waveforms recordings such as PSV were obtained and RI, PI and S/D ratio were calculated:

RI = PSV - end diastolic velocity/PSV

PI = PSV - end diastolic velocity/mean velocity

Patients were included in the study if they met the following criteria: (I) low risk pregnancy, (II) no evidence of fetal structural anomalies on the sonogram and normal neonatal anatomy, (III) accurate gestational age based on the last menstrual period with no difference from ultrasound parameters of more than 7 days, (IV) gestational age between 20 and 40 weeks, (V) normal fetal growth (more than 10th and less than 90th centile growth curves), (VI) normal uterine and umbilical arteries Doppler pattern by local reference values, (VII) availability of a detailed follow-up, (VIII) nonsmoker and nonalcoholic women, (IX) No history of hypertension, diabetes mellitus, autoimmune conditions, preeclampsia, abnormal vaginal discharge and bleeding, induced pregnancy, hydrops fetalis, and hormonal contraceptive agents intake such as LD.

Also patients who were developed the following conditions excluded from study: (I) congenital abnormalities, (II) oligohydramnios (amniotic fluid index (AFI) <5) according to Phelan's criteria [27], (III) biophysical profile <6, or estimated fetal weight outside the 90% normality range [28], (IV) inability to obtain MCA Doppler waveforms, (V) appearance of hypertension or preeclampsia during period after our assessment, (VI) loss of follow up, (VII) abnormal fetal biometry with an estimated fetal weight below the 10^th ^centile or higher than the 90^th ^centile in comparison with first trimester or early second trimester ultrasound findings (cases of IUGR, SGA and Large for gestational age), (VIII) only those who delivered a full term healthy baby with birth weight between the 10th and 90th percentiles for GA and gender were included for further data analysis, (IX) women with a notch and or a raised RI in a umbilical artery were excluded from the study as the risk of intrauterine growth restriction and pre-eclampsia [29].

Statistical analysis was performed using SPSS 14.0 (SPSS Inc., Chicago, IL, USA). Variables demonstrated as Mean ± SD. A value of *P *< 0.05 was considered statistically significant. Pearson correlation and Regression were used for evaluation of correlation between indices and gestational age. Reference ranges (90% range between 5^th ^and 95^th ^centiles) and the 95% confidence interval were constructed for each parameter and displayed in graphic form. Linear, quadratic and cubic regression models were fitted to estimate the relationship between fetal Doppler variables and gestational age (in weeks). The best fitting model for each variable was selected.

## Results

Altogether, 1037 women were evaluated during this study and among them only 978 (94.31%) were enrolled in final analysis and 59 patient were excluded due to loss of follow up (23 cases), abnormal umbilical Doppler resistive index (11 cases), inability to MCA Doppler waveforms measurement (8 cases), SGA (7 cases), low birth weight (4 cases), LGA (3 cases), and preeclampsia development (3 cases).

**Figure 1:**
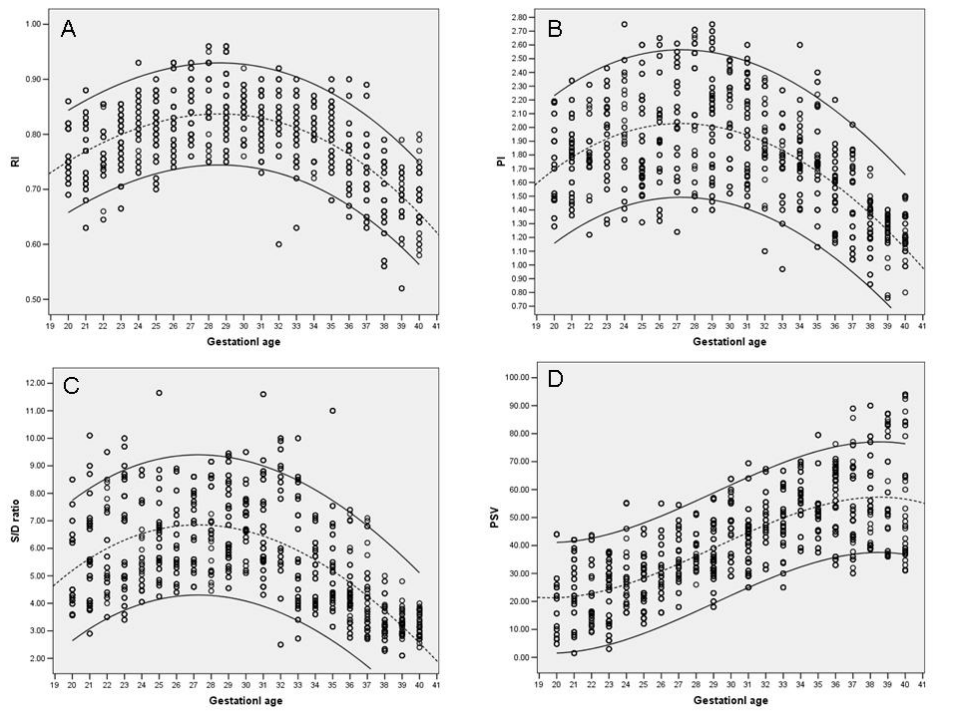
**(A) Individual measurements and calculated reference ranges for the resistive index (RI) in the MCA**. The standard boundaries include 90% of the normal patient population (Cubic R square = 0.386, P = 0.000). **(B) Individual measurements and calculated reference ranges for the pulsatility index (PI) in the MCA**. The standard boundaries include 90% of the normal patient population (Cubic R square = 0.340, P = 0.000). **(C) Individual measurements and calculated reference ranges for the systolic to diastolic ratio (S/D ratio) in the MCA**. The standard boundaries include 90% of the normal patient population (Cubic R square = 0.334, P = 0.000). **(D) Individual measurements and calculated reference ranges for the peak systolic velocity (PSV) in the MCA**. The standard boundaries include 90% of the normal patient population (Cubic R square = 0.535, P = 0.000).

The mean (± SD) birth weight was 3372.28 ± 551.43 g (2510–4980 g). The number of patients according to gestational age in weeks, patients' characteristics, mean and standard deviation for the MCA RI, PI, S/D ratio and PSV are shown in Table 1. Also values and nomograms of RI, PI, S/D ratio, and PSV, at 5, 50 and 95th percentile for each gestational age were shown respectively in Table 2 and Figure 1(A), (B), (C), and 1(D). The reference curve of the resistive index is characterized by parabolic pattern, showing a decrease of 0.76 to 0.67 at 20–40 weeks of gestation, and a peak RI value of 0.85 at 28 weeks of gestation. A similar pattern was observed for the pulsatility index (1.72 to 1.23 with a peak PI value 2.05 at 28 weeks of gestation) and S/D ratio (20^th ^week: 5.34, 40^th ^week: 3.16 and peak in 30^th ^week: 7.13). With regard to PSV, an increase of 20 to 54.42 cm/s with peak PSV value 60.85 in 39^th ^week was noted for the observation interval.

**Table 1:** Means and standard deviations of demographic and Doppler ultrasonographic findings in study population

***Gestational age***	***Number of patients***	***Age of patients***	***Patients gravidity***	***RI***	***PI***	***PSV***	***S/D ratio***
							
20	41	23.25 ± 3.41	1.31 ± 0.60	0.76 ± 0.04	1.72 ± 0.29	20.00 ± 12.23	5.34 ± 1.55
21	48	27.38 ± 6.34	1.79 ± 0.88	0.77 ± 0.06	1.79 ± 0.26	23.15 ± 12.69	5.81 ± 1.90
22	43	26.38 ± 6.37	1.69 ± 0.85	0.76 ± 0.05	1.82 ± 0.28	23.77 ± 11.69	5.91 ± 1.79
23	54	26.74 ± 6.70	1.81 ± 1.03	0.78 ± 0.04	1.94 ± 0.28	22.72 ± 11.02	6.31 ± 1.97
24	45	27.36 ± 5.95	2.00 ± 1.35	0.81 ± 0.05	1.94 ± 0.43	27.92 ± 11.38	6.21 ± 1.61
25	52	25.23 ± 5.21	1.81 ± 0.98	0.81 ± 0.05	1.90 ± 0.36	27.14 ± 9.20	6.38 ± 1.59
26	44	27.79 ± 4.51	1.74 ± 0.80	0.82 ± 0.05	1.95 ± 0.39	30.56 ± 10.14	6.36 ± 1.47
27	42	27.10 ± 5.31	2.10 ± 1.04	0.83 ± 0.04	2.03 ± 0.38	36.13 ± 9.37	6.69 ± 1.37
28	41	26.50 ± 3.62	1.60 ± 0.69	0.85 ± 0.07	2.05 ± 0.49	37.24 ± 6.60	6.95 ± 1.64
29	55	25.97 ± 5.56	1.60 ± 0.85	0.84 ± 0.05	2.02 ± 0.40	36.54 ± 9.70	6.87 ± 1.46
30	46	26.50 ± 4.47	1.70 ± 0.82	0.83 ± 0.04	1.98 ± 0.34	46.42 ± 11.16	7.13 ± 1.35
31	50	27.53 ± 7.70	1.57 ± 0.77	0.82 ± 0.04	1.97 ± 0.35	41.24 ± 10.14	6.60 ± 1.66
32	46	27.29 ± 5.97	2.06 ± 0.82	0.82 ± 0.07	1.92 ± 0.33	49.28 ± 9.77	7.09 ± 2.21
33	47	27.30 ± 4.78	1.52 ± 0.73	0.79 ± 0.06	1.76 ± 0.30	47.30 ± 10.73	5.84 ± 2.00
34	42	27.05 ± 6.02	1.81 ± 0.98	0.79 ± 0.04	1.79 ± 0.28	57.10 ± 9.29	5.10 ± 1.18
35	49	29.21 ± 4.65	2.26 ± 1.36	0.80 ± 0.05	1.75 ± 0.33	52.06 ± 9.61	5.33 ± 1.84
36	48	27.92 ± 5.62	2.33 ± 1.09	0.75 ± 0.05	1.54 ± 0.26	56.65 ± 12.2	4.31 ± 1.15
37	43	27.94 ± 5.05	1.63 ± 0.88	0.73 ± 0.07	1.43 ± 0.31	53.93 ± 16.34	4.12 ± 1.31
38	51	25.29 ± 5.65	1.53 ± 0.80	0.68 ± 0.06	1.25 ± 0.21	56.97 ± 15.91	3.22 ± 0.67
39	45	28.33 ± 5.66	2.27 ± 1.43	0.68 ± 0.05	1.23 ± 0.15	60.85 ± 18.96	3.22 ± 0.46
40	46	26.50 ± 2.33	1.63 ± 0.51	0.67 ± 0.04	1.23 ± 0.16	54.42 ± 23.48	3.16 ± 0.44

**Table 2:** 5, 50 and 95 percentiles values for Doppler waveforms indices according to gestational age

		**RI**			**PI**			**S/D ratio**			**PSV**		
					
		**5**	**50**	**95**	**5**	**50**	**95**	**5**	**50**	**95**	**5**	**50**	**95**
													
**Gestational age (Week)**	**20**	.69	.75	.86	1.28	1.71	2.19	3.56	4.5	8.5	4.8	21.5	44
	**21**	.65	.78	.86	1.37	1.82	2.27	3.28	5.51	9.6	4.0	22	42.
	**22**	.64	.75	.85	1.22	1.8	2.31	3.5	5.3	9.5	9	22	43.7
	**23**	.69	.78	.85	1.32	2	2.36	3.55	5.5	9.77	5.25	26	37.57
	**24**	.73	.81	.93	1.33	1.91	2.75	4.05	5.65	8.85	16	26	55
	**25**	.7	.81	.88	1.43	1.75	2.6	4.51	5.85	9.83	13.23	28	44.7
	**26**	.74	.81	.93	1.32	1.91	2.65	4.4	6	8.9	16	29	55
	**27**	.76	.82	.92	1.28	2.04	2.6	4.6	6.9	8.6	18.75	34	53.67
	**28**	.75	.84	.96	1.4	2.05	2.71	4.65	6.8	9.15	31	35.5	51
	**29**	.76	.83	.96	1.4	2.1	2.7	4.95	6.15	9.35	20	34.35	56.5
	**30**	.78	.84	.89	1.52	1.97	2.49	5.1	7.25	9.5	29	47.35	63.8
	**31**	.75	.82	.88	1.5	1.96	2.5	4.6	6	9.2	25	41.85	64
	**32**	.6	.83	.92	1.1	1.9	2.34	2.5	7.8	10	31.	49.1	67.4
	**33**	.66	.8	.89	1.08	1.78	2.21	2.95	5.5	9.51	26.75	46.4	65.16
	**34**	.74	.8	.86	1.4	1.73	2.54	3.63	5.1	7.18	38.18	58	70
	**35**	.68	.81	.9	1.13	1.74	2.4	3.15	4.72	11	37.5	52	79.5
	**36**	.65	.77	.88	1.16	1.58	2.05	2.81	4	7.22	33.9	61.5	70.55
	**37**	.63	.73	.89	1.04	1.37	2.02	2.7	3.75	7	30	50	89
	**38**	.56	.68	.79	.86	1.26	1.7	2.27	3	4.8	38.9	54	90
	**39**	.52	.69	.74	.76	1.27	1.4	2.1	3.23	3.9	36	53	87.2
	**40**	.64	.66	.73	.99	1.2	1.62	2.7	2.99	3.64	31.2	43	84.35

There was strong positive linear correlation between RI and PI (P = 0.000, R = 0.886), RI and S/D ratio (P = 0.000, R = 0.860), PI and S/D ratio (P = 0.000, R = 0.863) and negative linear correlation between PSV and PI (P = 0.001, R = -0.170) and PSV and S/D ratio (P = 0.012, R = -0.125).

## Discussion

Our findings demonstrated parabolic pattern for RI, PI, S/D and FHR curves and simple increasing pattern for PSV. Also, all of Doppler indices had linear correlation with gestational age. Our nomograms for the RI, PI, S/D ratio and PSV were compared to the results of Arduini et al. in Italy [30], Kurmanavicius et al. in Switzerland [31,32], Mari et al. in USA [17,33], Bahlmann et al. [10], Baschat et al. [5] in Germany, recently longitudinal study of Ebbing et al. [34] in Norway, and Rujiwetpongsron et al. [35] and Komwilaisak et al. in Thailand [36], as the references established to date.

In comparison of our reference RI curve with which published by Kurmanavicius et al [46], it was determined that reference limits during 24 to 40 weeks was lower about 0.6–1.1 for our curve. Like our study, Bahlmann et al. [14] found that reference curve for the RI was characterized by a parabolic pattern (18 weeks: 0.68; 28 weeks 0.8; 42 weeks: 0.61). We have demonstrated such a pattern for RI with some difference in ranges [see Figure 1(A)]. At last, our findings were in close relation with Bahlmann's findings. Rujiwetpongstorn's nomogram for MCA RI during 11–20 weeks demonstrated decreasing pattern without parabolic pattern [49]. Interestingly, our study period comes to complete this study period, it means that start point of our curve conjoined with the end point of Rujiwetpongstorn's RI curve.

The parabolic pattern of our PI curve is comparable to other studies [37,38]. The fall in the fetal MCA PI after 28^th ^week of gestation [see Figure 1(B)] was probably reflected a decreasing vascular resistance with increasing gestational age [37] or correlation with deoxyribonucleic acid production in fetal brain [38].

A comparison of the reference ranges established by this study with those of Arduini et al. [30] and Baschet et al. [5] showed almost identical parabolic pattern and reference values for the PI over the entire observation period. Conversely, the results reported by Bahlmann et al [10], Vyas et al [14], Mari et al [33],, Ebbing et al. [34], and Komwilaisak et al. [36] showed higher PI values, despite their curves pattern are uniform to our nomogram. The reasons for this discrepancy may be the 8- to 11-fold smaller number of patients included in Vyas and Mari studies and the different statistical methods and Doppler machines used may provide a further explanation for the marked deviations. Another important prerequisite for the deviation of accurate Doppler flow profiles from the MCA PI is that the pressure exerted by the ultrasound probe on the fetal head should be kept in a minimum; if this is not accomplished, extremely low end-diastolic flow velocities are measured, leading to the calculation of higher PI values [39]. As rationalized about RI, end point of Rujiwetpongstorn [35] curve on 20^th ^week was the same as our curve origin point.

There isn't more study about S/D ratio range and pattern, except Ertan's study that reported nomogram with decreasing slope toward end of gestation [40]. In our study, S/D ratio nomogram [Figure 1(C)] had parabolic pattern. Because the same factors were used to calculate the RI, PI and S/D ratio, similar pattern was expected. The principle reason for this pattern is decreasing blood flow resistancy with increasing of gestational age especially at the end of pregnancy that accompanying by blood flow volume increasing [23,33].

Our study demonstrated that the MCA PSV was increased during the second half of gestation [Figure 1(D)]. This finding was similar to that in the second half of pregnancy as reported by Bahlmann et al. [10] and Ebbing et al [34], demonstrated a continuous increase of MCA PSV over the period from 18 to 42 and 19 t0 41 weeks of gestation, respectively. Patterns were the same but values were higher for Bahlmann and Ebbing. Despite some differences in values, comparison of the PSV ranges in the MCA measured in this study with those reported by Mari et al [32] and Kurmanavicius et al [17] demonstrates good agreement. Explanations for these dissimilarities were the use of different statistical methods, curve analysis model, different sample sizes and more importantly sonologist skill and experience. Furthermore, an inverse correlation exists between peak systolic or mean blood flow velocities and the fetal hemoglobin or hematocrite concentration respectively, may interfere in results of every study that wasn't considered in major part of studies [17].

Our PSV curve interestingly is coming up to follow Rujiwetpongstorn's [35] presented curve and start point of our curve identical with end point of their curve. However, the variation of MCA indices in each gestational week was rather high.

A great number of DU reference curves have been obtained from various vessels of the fetal brain, yet primarily from the MCA. The results reported by these studies do not demonstrate any apparent difference between a longitudinal or cross-sectional study design [10,33].

This is clinically important to determine whether given MCA Doppler indices are normal or not, so normal MCA Doppler indices must be defined for each week of gestational age. Since these parameters may be varied among different population, population- specific nomograms may be needed. Although this study wasn't the first report of nomograms for MCA Doppler indices, it was an important step in assessment reliability and specificity of previous reported normal range especially in our population.

The strengths of present study were first, an adequate sample size. Unequal sample size for each gestational week did not discredit our data, because at least 20 pregnant women for each gestational age was adequately calculated and our enrolled population were more than two fold of this volume. Second, we were able to measure at all gestational age from 20 to 40 weeks. Third, we used only one experienced ultrasonologist to avoid inter-observer variation. At last, we used only one high resolution ultrasonography machine and one trans-abdominal transducer to avoid equipment's variation. As well as, our data had higher reliability based on this fact that all gestational age was established by careful history to identifying only patients with accurate date and confirmation by early ultrasound examination. Furthermore, all newborns were proved to have normal growth and having no structural abnormality.

During present study, the observations were included according to completed weeks. To avoid an artificial shift of data, the measurements have analyzed according to completed weeks of gestation. Therefore, the 5th and 95th percentiles do not cover up for the left shift in the results due to estimations based on completed weeks.

In some instances, differences were observed when comparing the reference ranges reported by various studies for the different parameters of the MCA. As noted previously for each Doppler index, the reasons are manifold and include: inconsistent choice of the wall filter, divergent size of the sample volumes, manual or automatic curve analysis, varying size of the patient populations, different mathematical methods for the calculation of reference ranges, variations in the application of the Doppler technologies, varying observation intervals.

The DU reference curves for the MCA described in this paper can be used in the clinical and scientific assessment of fetal hypoxemic, anemic disorders and intrauterine growth restriction [33], because these processes are identified by demonstration of low-impedance Doppler waveforms of the MCA [14].

## Conclusion

Our nomograms of MCA RI, PI, S/D ratio, PSV and FHR from 20^th ^to 40^th ^week is characterized by a typical parabolic pattern both for the RI, PI and S/D ratio of the MCA, with maximum values at 26^th^–31^st ^weeks of gestation and simple linear increasing pattern for PSV with maximum values at 38^th^–40^th ^weeks of gestation. There are no significant difference between our results and those reported by other authors in other regions among different populations (from East of Asia to West of Europe and America).

## Abbreviations

**DU**: Doppler ultrasonography; **MCA**: Middle cerebral artery; **RI**: Resistive index; **PI**: Pulsatility index; **S/D ratio**: Systolic to Diastolic ratio; **PSV**: Peak systolic velocity; **IUGR**: Pulsatility index; **SGA**: Small for gestational age; **BPD**: Biparietal diameter.

## Competing interests

The authors declare that they have no competing interests.

## Authors' contributions

MKT conceived the study, and carried out the Doppler ultrasound. NN participated in the design of the study, performed the statistical analysis, drafted the manuscript and revised of the manuscript. NS participated in Doppler ultrasonographic evaluation. NE participated in Doppler ultrasonographic evaluation. MT carried out clinical evaluation of pregnant women, recruiting them according to inclusion criteria and controlling newborns for excluding criteria. YT helped in statistical analyzing and revising of manuscript.

All authors read and approved the final manuscript.

## Pre-publication history

The pre-publication history for this paper can be accessed here:


